# Exploring the Prognostic Significance of *IL10* Variants and Their Mechanistic Regulation in Diabetic Nephropathy

**DOI:** 10.1111/jcmm.70819

**Published:** 2025-09-11

**Authors:** Neha Shukla, Shivani Kumari, Poornima Verma, Amisha Srivastava, Neelam Singh, Gyan Manjary Rao, Narayan Prasad, Sushil Gupta, Anshika Srivastava, M. S. Ansari, Soorya Janakiraman, Marey Baden, Olaf Wolkenhauer, Shailendra K. Gupta, Naveen Kumar Gautam

**Affiliations:** ^1^ Department of Urology and Renal Transplantation Sanjay Gandhi Post Graduate Institute of Medical Sciences Lucknow India; ^2^ Department of Nephrology Sanjay Gandhi Post Graduate Institute of Medical Sciences Lucknow India; ^3^ Department of Endocrinology Sanjay Gandhi Post Graduate Institute of Medical Sciences Lucknow India; ^4^ Department of Medical Genetics Sanjay Gandhi Post Graduate Institute of Medical Sciences Lucknow India; ^5^ Department of Systems Biology and Bioinformatics University of Rostock Rostock Germany

**Keywords:** CEBPA, diabetic nephropathy, genetic polymorphism, *IL10* gene polymorphism; rs1800871, rs1800896, KLF4, North Indian population, T2DM

## Abstract

*IL10* is a very effective anti‐inflammatory cytokine. *IL10* imbalance is linked to type 2 diabetes mellitus (T2DM) and also to renal hypertrophy, glomerular membrane thickening, and onset of diabetic nephropathy (DN). We aimed to investigate the association of *IL10* gene polymorphism (rs1800871T/C, rs1800896A/G) with DN and determine the influence of variants on its expression level and interaction with transcription factors. We genotyped 301 study subjects, comprising 75 DN, 126 T2DM patients, and 100 controls. All were analysed for biochemical assays and genotypic analysis by PCR‐RFLP and confirmed by Sanger sequencing. The haplotype analysis was calculated by Chi‐square test. mRNA expression and its correlation with variants were assessed by using RT‐PCR. Statistical analyses were done by using SPSS and GraphPad software. Screening of transcription factors with *IL10* gene variants was performed using the TRANSFAC database, and the variants impact on *IL10* molecular interactions was analysed. This study revealed that *IL10* gene polymorphism rs1800896 was significantly associated with DN. ‘CG’ and ‘TG’ haplotypes were significantly associated with DN. Expression levels of *IL10* were upregulated in DN patients. The genetic correlation study shows that *IL10* gene expression was downregulated in the rs1800871 alternate variant genotype (CC) while upregulated in the rs1800896 alternate variant genotype (GG). *In silico* analyses suggest that the binding affinity of transcription factor CEBPA decreases due to the rs1800871 alternate variant, while the affinity of KLF4 increases in the case of the rs1800896 alternate variant. Our *in silico* results correlated with *IL10* expression analysis in respective patient groups. Overall, our findings highlight the role of *IL10* gene polymorphism in DN progression in the North Indian population.

## 1 Introduction

Diabetic nephropathy (DN) is a secondary microangiopathic consequence of type 2 diabetes mellitus (T2DM) [1]. It is characterised in diabetic patients by the excretion of urine albumin, glomerular lesions, and a decline in the glomerular filtration rate (GFR) [2]. DN patients experience abnormalities in kidney structure and function, including mesangial enlargement, basement membrane thickening, nodular glomerulosclerosis, and tubulointerstitial fibrosis [3]. Any alterations within the kidney enhance immune cell activation and the release of pro‐inflammatory cytokines [4] associated with the progression of DN [5]. *IL10*, an anti‐inflammatory cytokine, reduces renal interstitial fibrosis by suppressing mesangial cell growth [6, 7]. *IL10* is generated by mesangial cells at the renal level, and its excessive rise induces mesangial cell proliferation, which causes alterations in the glomerular and interstitial tubules such as hypertrophy, glomerular membrane thickening, and albuminuria, that all contribute to renal failure [8]. Other studies also showed the relationship of an excessive higher concentration of serum *IL10* with the severity of DN [9]. Previous studies explored the association of *IL10* genes polymorphism rs1800871 and rs1800986 with the growth and development of T2DM and DN in various ethnicities. For example, *IL10* gene polymorphisms rs1800896 (A/G) linked with T2DM and DN risk in the Chinese and Southeast Iranian populations, but rs1800871 (T/C) did not show any association [10, 11, 12]. Similarly, rs1800896 (A/G) polymorphism was found to be associated with DN and T2DM in white Brazilians [13]. Tarabay and colleagues found the association of rs1800896 with T2DM in Asian and European ethnic groups, while rs1800871 only among African subjects [14]. Notably, the *IL10* gene polymorphism, rs1800871 and rs1800896, has been identified in the T2DM in North Indian populations [15]. Previous studies also suggest the association of these polymorphisms with the *IL10* expression level [16, 17]. However, there is a gap in knowledge regarding the association of both *IL10* gene polymorphism rs1800871 (T/C), rs1800896 (A/G) with DN progression in the North Indian population. Our study includes T2DM and DN patients and healthy controls from the North Indian population to explore the association of the *IL10* gene polymorphisms rs1800871 (T/C), rs1800896 (A/G) with DN, genotype correlation with mRNA expression level, and identification of transcription factor binding around the *IL10* gene polymorphism. A significant association of *IL10* gene polymorphism rs1800896 (A/G) with DN was observed. We also investigated that *IL10* gene polymorphisms rs1800871 (T/C) and rs1800896 (A/G) affect mRNA expression level. Our *in silico* analysis suggests that rs1800871 (T/C) and rs1800896 (A/G) regulate the expression level of *IL10* via transcription factors CEBPA and KLF4 respectively.

## 2 Methods

### 2.1 Subject Selection and Procurement of Samples for This Investigation

We enrolled 301 study subjects [healthy controls (HC) = 100, Type 2 diabetes mellitus patients (T2DM) = 126, and diabetic nephropathy patients (DN) = 75] from OPD of the Sanjay Gandhi Post Graduate Institute of Medical Sciences (SGPGIMS), Lucknow, India with the approval of the institutional ethics committee (IEC code: 2021‐331‐SRF‐EXP.44 PGVBE/189/2022). The sample size was calculated using power analysis with G*Power software (version 3.1.9.7), assuming a two‐sided 95% confidence interval and 80% statistical power. HC includes male and female individuals between the ages of 20 and 80 years, who have no history of diabetes or related complications. More specifically, patients with proteinuria preceding the onset of diabetes, along with other clinical concerns, such as heart disease and urinary tract infection, and an inadequate record, were excluded from the study. For the recruitment of the T2DM group, we followed ADA guidelines (American Diabetes Association, 2025) [18]. For the DN patient group, we followed standards defined in the KDIGO (Kidney Disease: Improving Global Outcomes, 2024) documents [19]. All the subjects in the study were selected from the North Indian ethnicity group. Two millilitres of peripheral blood samples were taken from all participants in a vial of EDTA for DNA extraction. Fresh 250 μL peripheral blood was taken in 750 μL TRI reagent BD solution for RNA extraction. One mL of blood was taken in non‐EDTA vials for serum separation for biochemical analysis. Serum was separated by centrifugation for 10 min at 4°C and 5000 rpm, and stored for later use at −20°C.

### 2.2 Anthropometric and Clinicopathological Characteristics

T2DM, DN patients, and healthy individuals were interviewed extensively for anthropometric (age and gender) and clinicopathological parameters such as systolic and diastolic blood pressure (SBP and DBP, respectively) were measured using a standardised proforma and cross‐verified by the study subject's medical records to reduce biases.

### 2.3 Biochemical Data

Fasting blood glucose, 2‐h post glucose value, glycated haemoglobin (HbA1c), Serum Creatinine Ratio Test (SCRT), and urine albumin excretion (UAE) data of patients were extracted from their medical records. The MDRD (modification of diet in renal disease) research equation was used to calculate the glomerular filtration rate (eGFR). The 24‐h UAE was gathered from the patients' accounts and validated using their medical records.

### 2.4 DNA Extraction and Genotypic Analysis

Genomic DNA was obtained from peripheral blood mononuclear cells utilising the salting out technique with minor changes [20, 21]. The DNA was quantified using a Nanodrop 1000 spectrophotometer and qualified using 0.8% agarose gel electrophoresis prior to being kept in a deep freezer at −80°C for future use. The Primer 3 program was used to create primers (Primer3web version 4.1.0) (Table 1) and validated using NCBI‐BLAST (https://blast.nlm.nih.gov) and UCSC *in silico* polymerase chain reaction (PCR) (https://genome.ucsc.edu/cgi‐bin/hgPcr), while NEB cutter V2 was used to select restriction enzymes (https://nc2.neb.com/NEBcutter2/?noredir). The PCR was conducted in a 25 μL reaction mixture, genomic DNA (50–100 ng), 10 pmol of primers, 10X buffer, 50 mM MgCl_2_, 200 mM dNTPs, and 0.5 U Taq DNA polymerase (Thermo Scientific), additionally MilliQ water. The Eppendorf master cycler was used for the initial denaturation at 94°C for 5 min, the annealing temperature at 55.6°C for 1 min, and extension at 72°C for 1 min for 40 cycles, with the final extension at 72°C for 5 min and hold at 4°C. On a 2% agarose gel, the length of the PCR product for rs1800871 was 206 and 451 bp for rs1800896. In a 20 μL reaction mixture, the PCR products were digested with Ms1I, HpyAV restriction enzymes (NEB) respectively overnight at 37°C and resolved on 3% agarose gel. The gel documentation system recorded the outcomes (BIO‐RAD).

**TABLE 1:** Selected *IL10* gene polymorphisms and the primer sequences for genotyping.

Gene	Reference SNP ID	SNP	Primer Sequence (5′‐3′)
*IL10*	rs1800871	T/C	Forward‐5′TCAACTTCTTCCACCCCATC3′ Reverse‐5′AGTGAGCAAACTGAGGCACA3′
	rs1800896	A/G	Forward‐5′TTCCCCAGGTAGAGCAACAC3′ Reverse‐5′CCATGACCCCTACCGTCTCT3′

### 2.5 Haplotype Analysis

The term ‘haplotype’ refers to the combination of alleles linked to different single nucleotide polymorphisms (SNPs) on the same chromosome. A haplotype analysis was performed for *IL10* gene polymorphisms (rs1800871 and rs1800896) using GraphPad Prism software. Chi square test was used in the study to investigate the association of haplotypes among T2DM, DN and HC.

### 2.6 Expression of *IL10*

Total RNA was extracted from blood samples collected in TRI Reagent BD and quantified on nanodrop (Eppendorf, Germany). For cDNA synthesis, Thermo Scientific's RevertAid First Strand cDNA synthesis kit was employed. The expression of *IL10* was measured using real‐time PCR (qPCR) with GAPDH as a reference. 1X Maxima SYBR Green master mix (Thermo Scientific), 25 ng of cDNA, 2.5 pmol specific primers (*IL10* F: 5‐GCCTTTAATAAGCTCCAAGAG‐3; *IL10* R: 5‐ATCTTCATTGTCATGTAGGC‐3, *GAPDH* F: TCGGAGTCAACGGATTT; R: CAACAATATCCACTTTACCAGA) and MilliQ water were used in the reaction mixture (10 mL). The reaction conditions were set to 1 cycle at 95**°**C for 10 min, then 40 cycles at 95**°**C for 10 s, 59**°**C for 30 s, 72**°**C for 30 s, and a final hold at 4**°**C. The relative quantification method (qPCR) was used for the expression analysis.

### 2.7 Statistical Analysis

SPSS software and Graph Pad Prism 5 were utilised for all statistical analyses. Data on anthropometry and biochemistry were presented as mean standard deviation. For comparison of biochemical parameters between two groups, independent sample t‐test was performed by using SPSS. We applied the Hardy–Weinberg equation by Chi‐square (*χ* ^2^) test. The sample size estimation was performed by using G*Power software. We analysed genotypic, allelic, and carriage rate frequencies in all groups and compared them by using Chi‐square and Fisher's exact test. Differences were considered statistically significant for *p*‐value < 0.05.

### 2.8 Screening of TF Binding Sites Around *IL10* Gene Polymorphisms

As both the *IL10* gene polymorphisms ‘rs1800871’ (position Chr1:206773289) and ‘rs1800896’ (position Chr1:206773552) are present within the 2 KB upstream of the 5'UTR region of *IL10* genes, we first identified experimentally validated transcription factor binding sites using the TRANSFAC database (BKL 2024.1 release) around the genomic locations of these two genetic variations. We later explored the ChIP‐Chip assay dataset and also used the TRANSFAC ‘MATCH’ algorithm to find potential regulatory factors that bind to the *IL10* rs1800871 and rs1800896 genomic variants investigated in this study.

### 2.9 Structure Model of Transcription Factors CEBP, KLF4 and *IL10* Promoter Fragments

Experimentally validated crystal structures of CEBP alpha‐DNA complex (PDB ID: 1NWQ) and KLF4 zinc finger DNA binding domain in complex with DNA (PDF ID: 6VTX) were used as templates for modelling and docking of CEBP and KLF4 with *IL10* promoter. First, the complex was analysed for the CEBP and KLF4 alpha amino acid residues involved in the interactions with DNA using ‘Intermolecular H Bond’ monitoring tool of the Biovia Discovery Studio 2022 software suite version 22.1.0.21297 (DS2022). These amino acid residues were grouped as potential ligand binding sites for further analysis.

### 2.10 3D Modelling of *IL10* Promoter Regions Containing rs1800871 and rs1800896 Polymorphism

We considered 10 nucleotides both upstream and downstream of rs1800871 genomic location. The sequence containing the T allele of SNP on the reverse strand (3′‐CGTGTCTCATAATGTAGTGG‐5′) is considered as wild type, whereas the alternate variant with a C allele at the same site is considered the alternate variant type. Similarly, for the rs1800896 variant, we considered the ‘A’ allele on the reverse strand (3′‐GATGAAGGGGAAGGGTTTCTT‐5′) as the wild type and the sequence with a ‘G’ allele as the alternate variant type. The initial 3D structures of wild‐type and alternate variant *IL10* promoter regions containing rs1800871 and rs1800896 variations were prepared using the ‘Build and Edit Nucleic Acid’ tool of DS2022.

### 2.11 Preparation of Protein and DNA Fragments for Molecular Docking Analysis

The transcription factor CEBP and KLF4 structures were subjected to the ‘Prepare Protein’ protocol of DS2022 for fixing various protein structure errors such as missing atoms in incomplete residues, missing loop regions, alternate conformations (disorder), non‐standard atom names, and incorrect protonation state of titratable residues. Both the transcription factors and the *IL10* promoter models were typed with a general purpose CHARMm force field [22] which has optimised parameters for the majority of organic molecules including protein and nucleic acids.

The ‘Smart minimizer’ algorithm of DS2022 was used for 5000 steps to generate the energy stable confirmations of CEBP, KLF4, and *IL10* promoter models suitable for molecular docking. The tolerance applied to the average RMS gradient during a cycle of minimisation was set to 0.1. The Generalised Born implicit solvent model with the dielectric constant value of 80 was used for the better approximation for the solvent effect on structure minimisation.

### 2.12 Molecular Docking of CEBP and KLF4 With Respective *IL10* Promoter Models

Molecular docking between transcription factors CEBP and KLF4 with *IL10* promoter models was performed by two independent tools including ZDOCK protocol available in the DS2022 and the pyDockDNA web server available at https://model3dbio.csic.es/pydockdna. While the ZDOCK tool is originally prepared for macromolecules (mainly for protein–protein interactions), the pyDockDNA is specifically designed to model protein‐DNA complexes.

In the ZDock protocol of DS2022, the optimised structure of CEBP and KLF4 was considered as receptor proteins, and the respective *IL10* promoter models as ligand molecules. The angular step size of 6 was used for finer conformational sampling of the ligand. The poses were filtered by defining the receptor binding site residues that were involved in DNA interactions in the original PDB file. A total of 2000 poses were saved and clustered using an RMSD cutoff of 10 Å from the cluster centre. A maximum number of clusters was set to 100. Further, the top pose from each cluster based on the ZRANK score was used for analysis and comparison with pyDockDNA results. For each the wild and alternate variant of *IL10* promoter fragments, the top 100 models based on the total binding energy were generated and complexed with CEBPA and KLF4 using the pyDockDNA server. Next, the top cluster poses from ZDOCK were superimposed on the complexes generated by the pyDockDNA server and only those with an RMSD less than 2 Å were selected for further analysis to explore the impact of genetic variants, rs1800871 and 1800896, on the binding affinities of transcription factors with the promoter.

## 3 Results

### 3.1 Anthro‐Clinicopathological Characteristics of Case and Control Groups

The anthro‐clinicopathological parameters for T2DM and DN are given in Table 2. The frequency of males in gender distribution is high in T2DM and DN as compared with HC and also shows a statistically significant association in the three groups HC/T2DM (*p* < 0.0001), HC/DN (*p* = 0.000), T2DM/DN (*p* = 0.026) (Table 2). SBP and DBP showed significant association with DN (*p* = < 0.0001, *p* = 0.003) as compared with controls, but DBP shows a non‐significant association with T2DM (*p* = 0.157) as compared with HC. Similarly, DBP shows a non‐significant association with DN compared with T2DM (*p* = 0.100) (Table 2). Blood glucose (fasting and postprandial) was significantly higher in T2DM and DN as compared to controls (*p* = < 0.0001). In the other group, fasting sugar levels show a significant association (*p* = 0.012) but postprandial sugar levels were found to be not significant (*p* = 0.965) with DN compared to T2DM. HbA1c and creatinine levels were also considerably higher (*p* < 0.0001) in DN patients compared to controls and T2DM (Table 2). Further, 24 h' urine albumin excretion showed a higher significant association (*p* < 0.0001) in DN as compared with HC and T2DM (*p* < 0.0001) (Table 2).

**TABLE 2:** Association of anthro‐clinicopathological and biochemical parameters with type 2 diabetes mellitus, diabetic nephropathy, and healthy individuals (controls).

Anthro‐clinicopathological and biochemical parameters	Control mean ± SD (*n* = 100)	T2DM patients mean ± SD (*n* = 126)	DN patients mean ± SD (*n* = 75)	Control versus T2DM *p*	Control versus DN *p*	T2DM versus DN *p*
Men	20 (20%)	82 (65%)	60 (80%)	**< 0.0001**	**< 0.0001**	**0.026**
Women	80 (80%)	44 (35%)	15 (20%)			
Age (years)	48.1 ± 9.3	53 ± 10	56.04 ± 9.4	0.160	0.944	0.179
Systolic blood pressure (SBP) (mmHg)	116.2 ± 7.3	127.7 ± 17	137.5 ± 21	**< 0.0001**	**< 0.0001**	**0.012**
Diastolic blood pressure (DBP) (mmHg)	75.6 ± 8.9	75 ± 11	80.2 ± 13	0.157	**0.003**	0.100
Fasting blood sugar (mg/dL)	89.5 ± 10	142.3 ± 43	178.4 ± 58	**< 0.0001**	**< 0.0001**	**0.012**
Postprandial (mg/dL)	97.7 ± 18	239.7 ± 73	234.5 ± 71	**< 0.0001**	**< 0.0001**	0.965
HbA1C (%)	5.4 ± 0.4	7.96 ± 1.3	8.3 ± 1.6	**< 0.0001**	**< 0.0001**	**0.053**
Creatinine (mg/dL)	0.9 ± 0.1	1.0 ± 0.2	2.8 ± 0.1	0.251	**< 0.0001**	**< 0.0001**
eGFR (mL/min/1.73 m^2^)	82.2 ± 19	88.3 ± 24	38.8 ± 22.9	0.118	0.059	0.771
24‐h urine albumin excretion (mg/g)	17.1 ± 2.6	18.8 ± 2.8	323.4 ± 173	0.334	**< 0.0001**	**< 0.0001**

*Note:* Bold values represent *p* < 0.05 at 95% Confidence interval.

Abbreviations: DN, diabetic nephropathy; eGFR, estimating glomerular filtration rate; HbA1c, glycated haemoglobin; SD, standard deviation; T2DM, type 2 diabetes mellitus; UAE, urine albumin excretion.

### 3.2 Genotyping of *IL10* rs1800871 (T/C) and rs1800896 (A/G)

**FIGURE 1:**
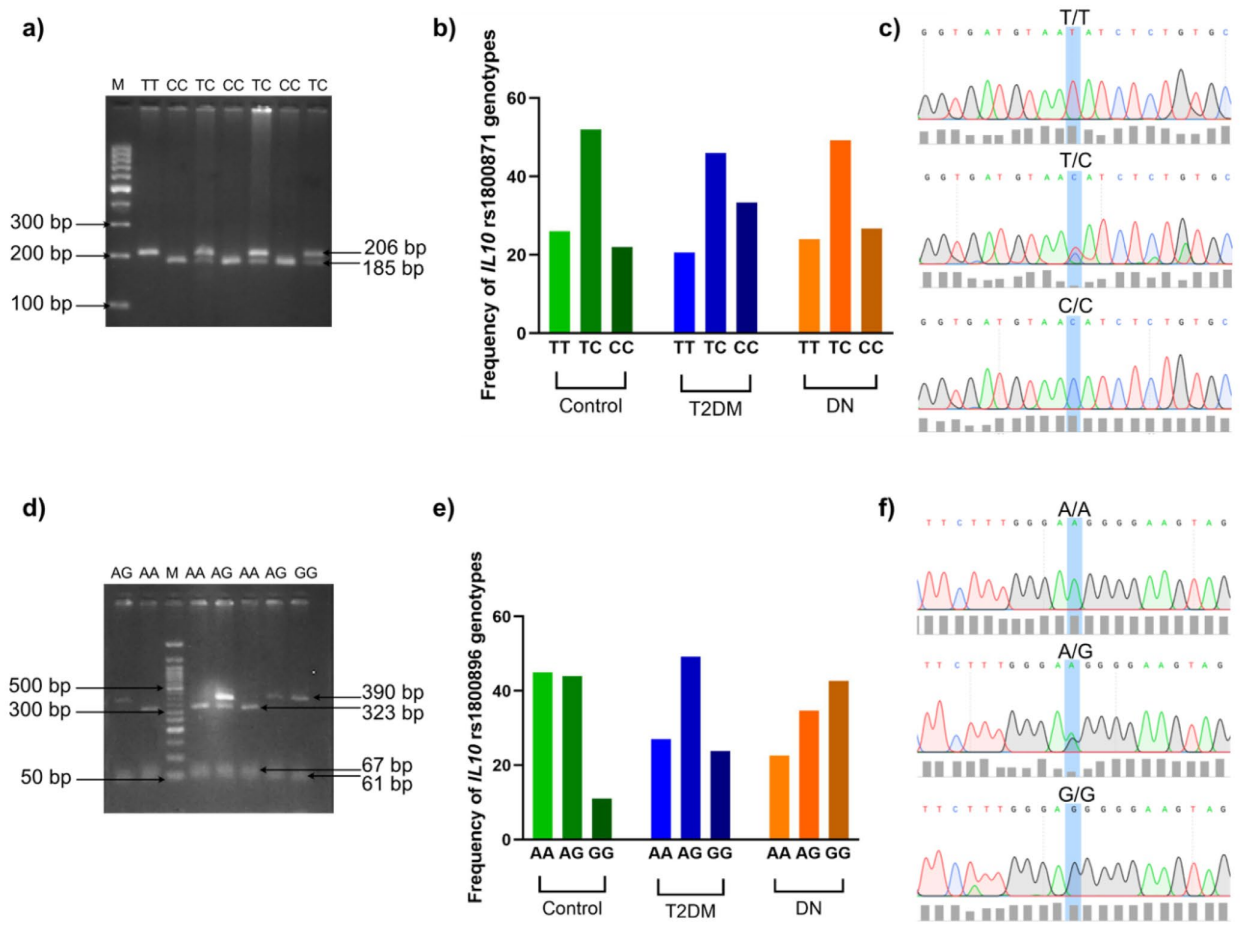
*IL10* gene variants rs1800871 and rs1800896 genotyping. (a) 3% Agarose gel showing different genotypes of *IL10* rs1800871 T/C, Lane 1 shows marker (100–1000 bp), lane 2 shows TT (206 bp), lane 3, 5, 7 shows CC (185, 21 bp), lane 4,6,8 shows TC (206, 185, 21 bp) digest with MS1I. (b) Frequency of *IL10* gene variant rs1800871 in the patient cohort. (c) Chromatogram confirms the presence of all IL10 rs1800871 genotypes. (d) 3% Agarose gel showing different genotypes of *IL10* rs1800896 A/G, Lane 3 shows marker (50–1000 bp), lane 2,4 shows AA (323, 67, 61 bp), lane 1,5,7 shows AG (390, 323, 67, 61 bp), lane 8, shows GG (390, 61 bp), digest with Hpyav. (e) Frequency of *IL10* gene variant rs1800896 in the patient cohort. (f) Chromatogram confirms the presence of all *IL10* rs1800896 genotypes.

Genotyping was done by amplifying *IL10* gene polymorphism rs1800871 T/C and rs1800896 A/G by PCR. The PCR product of these polymorphisms was 206 and 451 base pairs (bp) respectively. Ms1I digestion resulted in 206, 185, and 21 bp fragments; the ‘TT’ genotype was represented by 206 bp; ‘TC’ genotype by 206, 185, and 21 bp; and ‘CC’ genotype by 185 and 21 bp (Figure 1a). HpyAv digestion resulted in 390, 323, 67, and 61 bp fragments; the ‘AA’ genotype was represented by 323, 67, and 61 bp; ‘AG’ genotype by 390, 323, 67, and 61; and the ‘GG’ genotype by 390 and 61 bp (Figure 1d). The purified PCR amplicon of *IL10* gene polymorphisms rs1800871 and rs1800896 were further validated in randomly selected samples using Sanger sequencing (Figure 1c,f).

### 3.3 Association of *IL10* rs1800871 (T/C) and rs1800896 (A/G) Gene Polymorphism With T2DM and DN Patient Groups

The genotypic and allelic frequency distributions, as well as the carriage rates of the *IL10* polymorphism were calculated in 100 healthy controls, 126 T2DM, and 75 DN cases. The Carriage rates refer to the proportion of individuals carrying at least one copy of the alternate variant allele (either in heterozygous or homozygous form), which helps in understanding the distribution of risk alleles within the study groups. We applied the Hardy–Weinberg equilibrium (HWE) equation to assess the genotype distributions of *IL10* polymorphisms rs1800871 (T/C) and rs1800896 (A/G) across all study groups. For rs1800871 (T/C), the genotype distribution in the control group was ‘TT’: 26, ‘TC’: 52, and ‘CC’: 22; in the T2DM group, ‘TT’: 26, ‘TC’: 58, and ‘CC’: 42; and in the DN group, ‘TT’: 18, ‘TC’: 37, and ‘CC’: 20. Similarly, for rs1800896 (A/G), the genotypes distribution in the control group were ‘AA’: 45, ‘AG’: 44, and ‘GG’ 11; in the T2DM group, ‘AA’: 34, ‘AG’: 62, and ‘GG’: 30; and in the DN group, ‘AA’: 17, ‘AG’: 26, and ‘GG’: 32. The genotype frequencies in all groups were found in Hardy–Weinberg equilibrium (Table 3). In the *IL10* rs1800871 (T/C) genotypic frequencies of ‘TC’ and ‘CC’, ‘C’ allele and carriage rate were found higher but statistically non‐significantly associated in T2DM and DN patient groups compared with HC. In *IL10* rs1800896 (A/G) the genotypic frequency of the ‘GG’ genotype was found to significantly higher in the T2DM and DN patients with odd ratio up to 3.61 (*p* = 0.002) and 7.70 (*p* = 0.0001) in comparison to healthy control. While comparing ‘GG’ genotype in DN, we found higher frequency which was statistically significant with the odd ratio 2.133 (*p* = 0.058) as comparison to T2DM. In addition, heterozygous ‘AG’ genotype frequency was increased in T2DM and decrease in DN patients with odds ratio 1.86 (*p* = 0.053) and 1.56 (*p* = 0.267) as compared to HC. Moreover, in DN patient ‘AG’ genotype was decreased as compared with T2DM with the odds ratio 0.838 (*p* = 0.704). Similarly, the ‘G' allele frequency was significantly higher in both the T2DM group (odds ratio = 1.90, *p* = 0.001) and the DN group (odds ratio = 3.00, *p* = 0.0001) when compared to healthy controls. Additionally, the DN group showed a higher ‘G' allele frequency compared to the T2DM group (odds ratio = 1.59, *p* = 0.030), suggesting a progressive increase in allele frequency from controls to T2DM to DN. We found the carriage rate frequency association significantly higher in T2DM (A^+^/A^−^: odd ratio 2.52 and *p* = 0.014; G^+^/G^−^: odds ratio 0.45 and *p* = 0.0052) and DN patient groups (A^+^/A^−^: odd ratio 6 and *p* = 0.0001; G^+^/G^−^: odds ratio 0.35 and *p* = 0.002) compared to HC. The carriage rate frequency was also higher in DN compared to T2DM group only for A^+^/A^−^ allele (odd ratio 2.38 and *p* = 0.007) while no association was observed for the G^+^/G^−^ allele (odd ratio 0.793 and *p* = 0.615). In summary, we observed higher frequency of ‘GG' genotype, ‘G' allele and carriage rate in the T2DM and DN patients compared to HC from North Indian Population. The comparison of genotypic, allelic frequencies and carriage rate of both the *IL10* gene polymorphism in various studied groups are also summarised in the Table 4.

**TABLE 3:** Assessment of Hardy–Weinberg equilibrium (HWE) of studies SNP in healthy controls, T2DM and DN.

Gene/Reference ID/SNP	Controls (*n* = 100) distribution	HWE *χ* ^2^ *p*	Type 2 diabetes mellitus patients (*n* = 126) distribution	HWE *χ* ^2^ *p*	Diabetic nephropathy (*n* = 75) distribution	HWE *χ* ^2^ *p*
*IL10* rs1800871 (T/C)	TT (26)	*χ* ^2^ (0.174) *p* (0.676)	TT (26)	*χ* ^2^ (0.420) *p* (0.517)	TT (18)	*χ* ^2^ (0.012) *p* (0.912)
	TC (52)		TC (58)		TC (37)	
	CC (22)		CC (42)		CC (20)	
*IL10* rs1800896 (A/G)	AA (45)	*χ* ^2^ (0.002) *p* (0.96)	AA (34)	*χ* ^2^ (0.017) *p* (0.895)	AA (17)	*χ* ^2^ (5.787) *p* (0.016)
	AG (44)		AG (62)		AG (26)	
	GG (11)		GG (30)		GG (32)	

**TABLE 4:** Genotypic, allelic frequencies and carriage rate of *IL10* gene polymorphism rs1800871 (T/C), rs1800871 (A/G) in T2DM (*n* = 126), DN patients (*n* = 75), and healthy individuals (controls) (*n* = 100).

*IL10* gene polymorphism									
rs1800871 (T/C)					rs1800896 (A/G)				
Genotype/Alleles	Controls (%)	Type 2 diabetes mellitus patients (%)	*p*	Odd Ratio (OR) (95% CI)	Genotype/Alleles	Controls (%)	Type 2 Diabetes mellitus patients (%)	*p*	Odd ratio (OR) (95% CI)
**Genotypic frequencies**					**Genotypic frequencies**				
TT	26 (26)	26 (20.6)	—	1.0 (Ref.)	AA	45 (45)	34 (27)	—	1.0 (Ref.)
TC	52 (52)	58 (46.0)	0.866	1.11 (0.58–2.10)	AG	44 (44)	62 (49.2)	0.053	1.86 (1.04–3.40)
CC	22 (22)	42 (33.4)	0.129	1.90 (0.92–4.04)	GG	11 (11)	30 (23.8)	**0.002**	3.61 (1.59–7.85)
**Alleles frequencies**					**Alleles frequencies**				
T	104 (52)	110 (43.6)	0.088	1.39 (0.95–2.01)	A	134 (67)	130 (51.6)	**0.001**	1.90 (1.30–2.77)
C	96 (48)	142 (56.4)			G	66 (33)	122 (48.4)		
**Carriage rate**					**Carriage rate**				
T+	78 (78)	84 (66.6)	0.074	1.77 (0.98–3.22)	A+	89 (89)	96 (76.2)	**0.014**	2.52 (1.22–5.50)
T−	22 (22)	42 (33.4)			A−	11 (11)	30 (23.8)		
C+	74 (74)	100 (79.4)	0.345	0.74 (0.40–1.36)	G+	55 (55)	92 (73)	**0.0052**	0.45 (0.264–0.787)
C−	26 (26)	26 (20.6)			G−	45 (45)	34 (27)		

Genotype/Alleles	Controls (%)	Diabetic nephropathy patients (%)	*p*	Odd ratio (OR) (95% CI)	Genotype/Alleles	Controls (%)	Diabetic nephropathy patients (%)	*p*	Odd ratio (OR) (95% CI)
**Genotypic frequencies**					**Genotypic frequencies**				
TT	26 (26)	18 (24)	—	1.0 (Ref.)	AA	45 (45)	17 (22.6)	—	1.0 (Ref.)
TC	52 (52)	37 (49.3)	0.999	1.02 (0.47–2.12)	AG	44 (44)	26 (34.7)	0.267	1.56 (0.728–3.30)
CC	22 (22)	20 (26.7)	0.664	1.31 (0.56–3.13)	GG	11 (11)	32 (42.7)	**0.0001**	7.701 (3.048–19)
**Alleles frequencies**					**Alleles frequencies**				
T	104 (52)	73 (48.6)	0.589	1.14 (0.75–1.74)	A	134 (67)	60 (40)	**0.0001**	3 (1.95–4.72)
C	96 (48)	77 (51.4)			G	66 (33)	90 (60)		
**Carriage rate**					**Carriage rate**				
T+	78 (78)	55 (73.3)	0.480	1.28 (0.63–2.60)	A+	89 (89)	43 (57.4)	**0.0001**	6 (2.75–12.76)
T−	22 (22)	20 (26.7)			A−	11 (11)	32 (42.6)		
C+	74 (74)	57 (76)	0.860	0.898 (0.45–1.76)	G+	55 (55)	58 (77.3)	**0.002**	0.35 (0.18–0.69)
C−	26 (26)	18 (24)			G−	45 (45)	17 (22.7)		

Genotype/Alleles	Type 2 diabetes mellitus patients (%)	Diabetic nephropathy patients (%)	*p*	Odd ratio (OR) (95% CI)	Genotype/Alleles	Type 2 diabetes mellitus patients (%)	Diabetic nephropathy patients (%)	*p*	Odd ratio (OR) (95% CI)
**Genotypic frequencies**					**Genotypic frequencies**				
***IL10*rs1800871 (T/C)**					***IL10*rs1800896 (A/G)**				
TT	26 (20.6)	18 (24)	—	1.0 (Ref.)	AA	34 (27)	17 (22.6)	—	1.0 (Ref.)
TC	58 (46.0)	37 (49.3)	0.853	0.92 (0.43–1.88)	AG	62 (49.2)	26 (34.7)	0.704	0.838 (0.414–1.77)
CC	42 (33.4)	20 (26.7)	0.413	0.68 (0.32–1.48)	GG	30 (23.8)	32 (42.7)	**0.058**	2.133 (1.017–0.45)
**Alleles frequencies**					**Alleles frequencies**				
T	110 (43.6)	73 (48.6)	0.352	0.81 (0.54–1.22)	A	130 (51.6)	60 (40)	**0.030**	1.59 (1.06–2.38)
C	142 (56.4)	77 (51.4)			G	122 (48.4)	90 (60)		
**Carriage rate**					**Carriage rate**				
T+	84 (66.6)	55 (73.3)	0.347	0.72 (0.39–1.39)	A+	96 (76.2)	43 (57.4)	**0.007**	2.38 (1.26–4.33)
T−	42 (33.4)	20 (26.7)			A−	30 (23.8)	32 (42.6)		
C+	100 (79.4)	57 (76)	0.599	1.21 (0.59–2.33)	G+	92 (73)	58 (77.3)	0.615	0.7931 (0.39–1.54)
C−	26 (20.6)	18 (24)			G−	34 (27)	17 (22.7)		

*Note:* Bold values represent *p* < 0.05 at 95% confidence interval.

### 3.4 Haplotype Analysis in the Combinations *IL10* ‐rs1800871 (T/C) and rs1800896 (A/G) Polymorphism

Haplotype analysis was performed in three groups: HC and T2DM cases, HC and DN cases, and T2DM and DN cases. In the haplotype analysis of *IL10* gene polymorphism, rs1800871(T/C) and rs1800896(A/G) four distinct haplotype combinations, ‘CA’, ‘CG’, ‘TG’, and ‘TA’ were found in all three groups. The ‘CA’, ‘CG’, and ‘TG’ (‘TA’ is reference wild type) haplotype was associated with T2DM in comparison with HC (*p* = 0.0046, *p* = < 0.0001, *p* = 0.0003). Interestingly, these ‘CG’ (*p* = < 0.0001) and ‘TG’ (*p* = < 0.0001) haplotypes showed significant association in DN as compared with HC (Table 5).

**TABLE 5:** Haplotype analysis of *IL10* gene polymorphism T/C (rs1800871) and A/G (rs1800896) in type 2 diabetes mellitus (T2DM) (*n* = 126), diabetic nephropathy (DN) patients (*n* = 75), and healthy individuals (controls) (*n* = 100).

Haplotypes	Control *n* (%)	T2DM *n* (%)	*p*	Odds ratio [95% CI]
TA	59 (29.5)	35 (13.9)	—	1.0 (Ref.)
CA	75 (37.5)	95 (37.7)	**0.0046**	2.135 (1.284–3.524)
CG	21 (10.5)	47 (18.6)	**< 0.0001**	3.773 (1.957–7.343)
TG	45 (22.5)	75 (29.8)	**0.0003**	2.810 (1.616–4.835)

Haplotypes	Control *n* (%)	DN *n* (%)	*p*	Odds ratio [95% CI]
TA	59 (29.5)	19 (12.7)	—	1.0 (Ref.)
CA	75 (37.5)	41 (27.3)	0.1154	1.698 (0.9151–3.194)
CG	21 (10.5)	36 (24)	**< 0.0001**	5.323 (2.500–11.26)
TG	45 (22.5)	54 (36)	**< 0.0001**	3.726 (1.896–7.157)

Haplotypes	T2DM *n* (%)	DN *n* (%)	*p*	Odds ratio [95% CI]
TA	35 (13.9)	19 (12.7)	—	1.0 (Ref.)
CA	95 (37.7)	41 (27.3)	0.4951	0.7950 (0.4039–1.564)
CG	47 (18.6)	36 (24)	0.3761	1.411 (0.7012–2.776)
TG	75 (29.8)	54 (36)	0.4142	1.326 (0.6889–2.570)

*Note:* Bold numerals in table show significant association.

Abbreviations: CI, confidence interval; OR, odds ratio.

### 3.5 Expression of *IL10* Gene and Its Correlation With Various Genotypes

**FIGURE 2:**
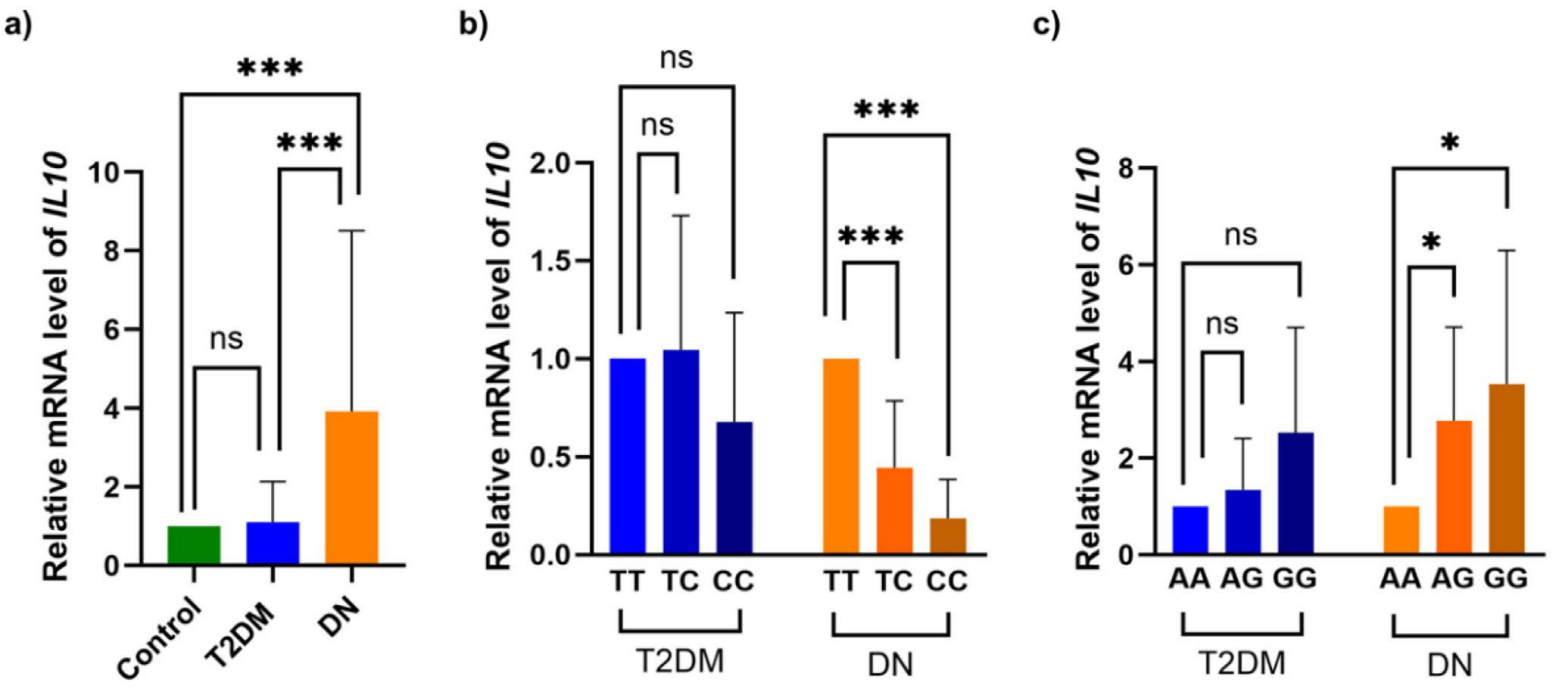
*IL10* expression level and its correlation with genotypes. (a) *IL10* mRNA expression level in T2DM and DN compared to HC. (b) *IL10* expression level in various TT, TC, CC genotype in rs1800871 variants within T2DM and DN patients. ‘TT’ genotype is considered as a wild‐type. (c) *IL10* expression level in various AA, AG, GG genotype in rs1800896 variants within T2DM and DN patients. ‘AA’ genotype is considered as a wild‐type. Statistical significance is indicated as *P < 0.05 and ***P < 0.001.

The level of mRNA expression of *IL10* in patients and the controls group was measured by real‐time PCR. The relative mRNA expression was non‐significant in T2DM but significantly upregulated in DN (*p* = 0.0004) as compared to HC (Figure 2a). Likewise, there is a marked upregulation in relative mRNA expression (*p* = 0.0004) in the DN group when compared to the T2DM (Figure 2a). We further analysed and compared the *IL10* gene expression level in various rs1800871 and rs1800896 associated genotypes within the T2DM and DN patient groups.

We could not find significant changes in the *IL10* expression level in the alternate variant ‘TC’ and ‘CC’ genotypes as compared to the wild‐type ‘TT’ for rs1800871 and also for the alternate variant ‘AG’ and ‘GG’ genotypes as compared to the wild‐type ‘AA’ for rs1800896 in the T2DM group (Figure 2b,c). Further investigation shows that the level of *IL10* was significantly downregulated in the alternate variant genotypes (‘TC’: *p* = 0.0008, ‘CC’: *p* = < 0.0001) of rs1800871, while upregulated in the alternate variant genotypes (‘AG’: *p* = 0.0286, ‘GG’: *p* = 0.0285) of DN patients carrying rs1800896 variants compared to their respective wild‐type genotypes (‘TT’, ‘AA’) (Figure 2b,c).

### 3.6 Identification of Transcription Factors Bound to the *IL10* Promoter Around rs1800871 and rs1800896 Genetic Variants

**FIGURE 3:**
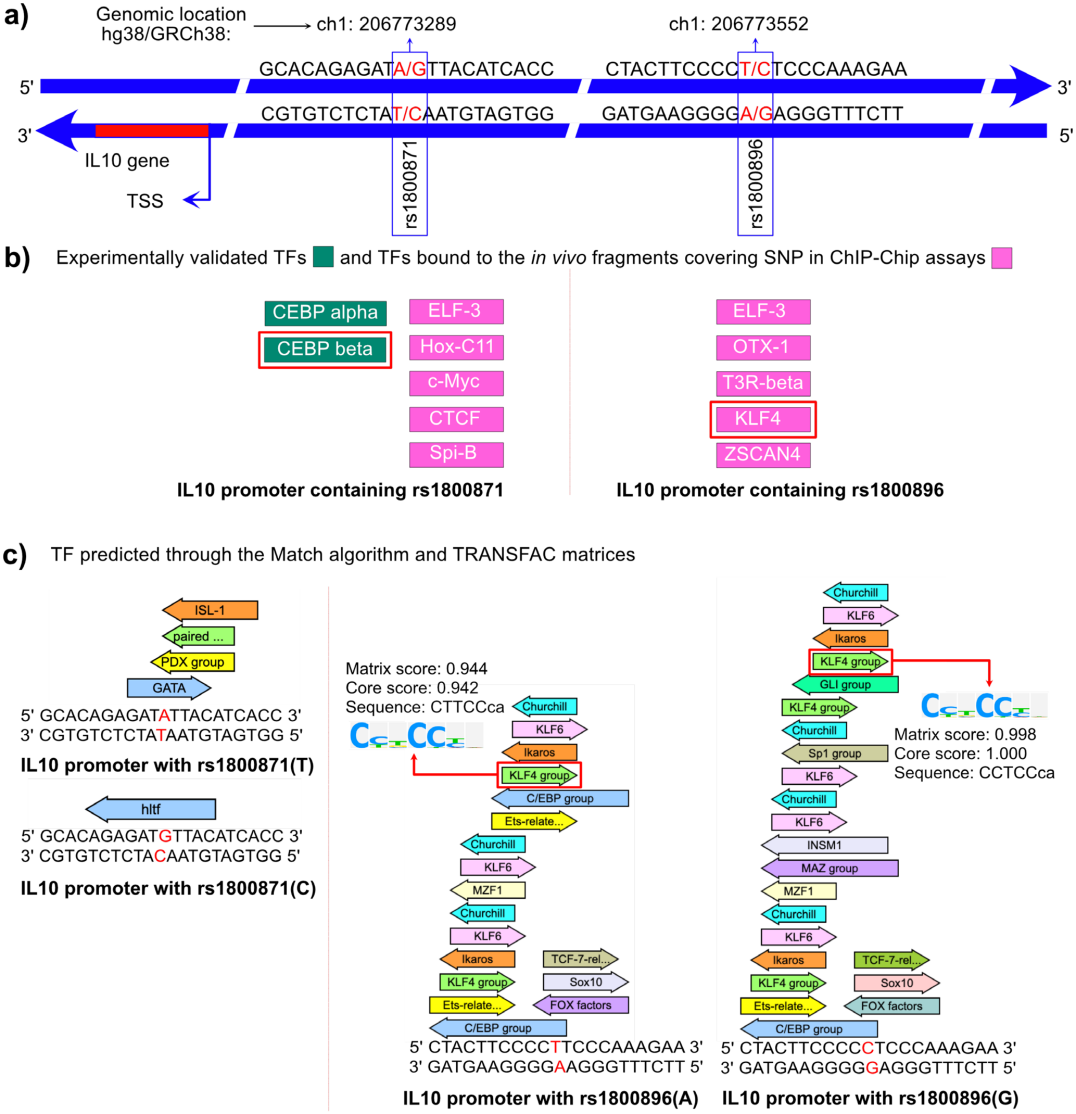
Identification of transcription factors (TF) that are bound on and around the IL10 rs1800871 and 1800896 genomic locations. (a) Schematic diagram of *IL10* gene and its promoter region. Both the DNA strands are highlighted as blue arrow. *IL10* gene (red colour) is present on the negative strand. Two genetic variations (rs1800871 and rs1800896) investigated here are highlighted together with 10 bases up and downstream as nucleotide sequences on the promoter along with their genomic locations. Both wild and alternate variant nucleotides are shown in red colour. (b) Experimentally validated TFs (green boxes) and TFs bound to the in vivo fragments covering SNPs in the ChIP‐Chip assays (pink boxes) of *IL10* promoters are shown. TFs that are further investigated with wild and alternate variant promoter fragments of both the SNPs are enclosed with red boxes. (c) TFs predicted through the Match algorithms in the *IL10* promoter fragments (10 bases up and downstream of SNPs) are shown for both wild type and alternate variants of rs1800871 and rs1800896.

First, we screened the TRNASFAC database (BKL 2024.1 release) for experimentally validated transcription factors that are bound to the promoter region of the *IL10* gene. The TRNASFAC analysis indicates that the rs1800871 genetic variants (Genomic location hg38/GRCh38:Ch1 206773289) are part of CCAAT/enhancer‐binding protein (CEBP) alpha and beta transcription binding sites (Ch1:206773284–206773303) which transactivate the *IL10* gene. This variation is located 863 nucleotides upstream of the transcription start site (TSS). We could not find any experimentally validated transcription factor binding sites on the *IL10* promoter fragment that include rs1800896 (Genomic location hg38/GRCh38:Ch1 206773552) (Figure 3a).

Next, we explored potential transcription factors using the ChIP‐Chip assay dataset available on TRANSFAC. We found five different TFs bound to the in vivo fragments of different lengths containing rs1800871 and rs1800896 genomic locations (Figure 3b).

Finally, we also used the MATCH tool [23] and the ‘vertebrate‐non‐redundant’ matrix profile available on the TRANSFAC to predict the TFs that may bind on/around rs1800871 and rs1800896 genomic locations (10 bases up and downstream of the variation) for both wild‐type and alternate variant promoter sequences. Although we could not find any common TFs for the genomic location rs1800871 with three different analyses carried out in this work, that is, screening of (1) Experimentally validated TFs; (2) TFs present in the ChIP‐Chip assay dataset; and (3) Prediction using the TRANSFAC MATCH algorithm, we indeed found the TF Krüppel‐like factor 4 (KLF4) common in the ChIP‐Chip assay dataset and the MATCH algorithm analyses of the rs1800896 genomic location (Figure 3c). Upregulation of TF KLF4 was also positively correlated with the *IL10* gene expression in previous studies [24, 25]. Using the MATCH algorithm, we found that both the core and matrix similarity scores increase for the rs1800896 alternate variant ‘G’ allele in comparison to the wild‐type ‘A’ allele.

### 3.7 Effect of *IL10* Gene Polymorphism rs1800871 and rs1800896 on Transcription Factor Interactions

To further explore the impact of *IL10* gene polymorphism on the binding affinity with selected TFs, we performed molecular docking analysis. For this, we prepared 3D structures of *IL10* promoter fragments (dsDNA with 10 nucleotides in both up and downstream directions of the variants) containing ‘T’ allele (wild type) and alternative ‘C’ allele (alternate variant type) for the rs1800871 and similarly, ‘A’ allele (wild type) and ‘G’ allele (variant type) for the rs1800896 variants. The DNA and protein interaction analyses were performed by two separate molecular docking algorithms ZDOCK [26] and pyDockDNA [27]. While the ZDOCK algorithm is primarily developed to study protein–protein interactions, some of the previous studies also proved the effectiveness of ZDOCK for protein‐DNA interactions [28, 29, 30].

**FIGURE 4:**
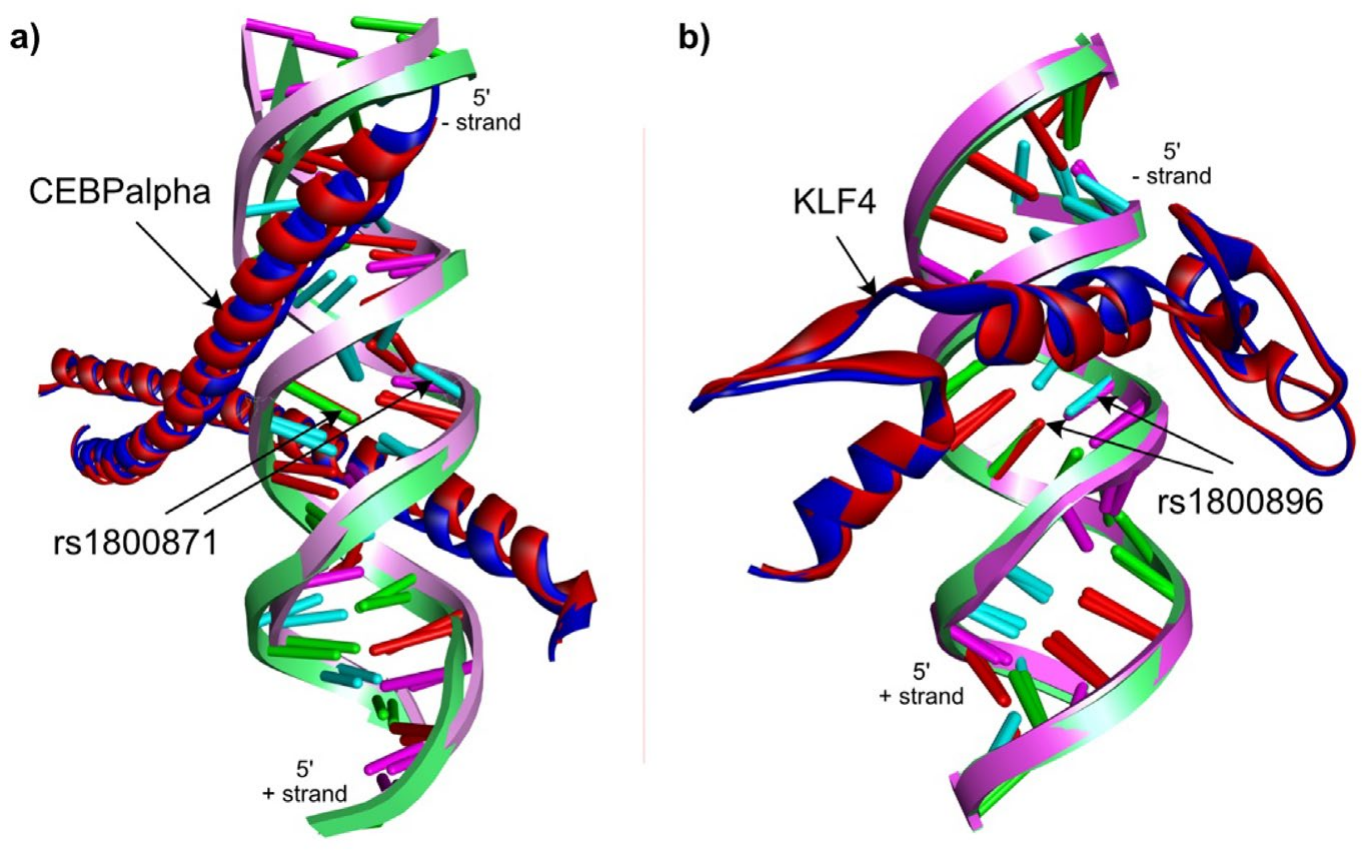
Best interaction pose of *IL10* promoter fragments with associated transcription factors. (a) The superimposed structures of wild and alternate variant *IL10* dsDNA fragment with rs1800871 genomic variant in complex with CEBPA transcription factor are shown. (b) The superimposed structures of wild and alternate variant *IL10* dsDNA fragment with rs1800896 genomic variant in complex with KLF4 transcription factor are shown. Wild‐type dsDNA fragments are highlighted in green. The transcription factors in complex with the wild‐type dsDNA fragments are shown in blue and with the alternate variant dsDNA fragments are shown in red.

The top interaction poses selected after analysing the ZDOCK and pyDockDNA results indicate that both the wild and alternate variant *IL10* dsDNA fragments containing rs1800871 variants interact with the homodimeric form of CEBP alpha (CEBPA) transcription factor (Figure 4a). However, we found that the best poses for the wild‐type *IL10 dsDNA* fragment (‘T’ allele) with CEBPA were slightly more stable (binding energy: −333.457 kcal/mol) in comparison to the best binding pose we identified for the alternate variant dsDNA fragment (‘C’ allele) (binding energy: −331.969 kcal/mol). We also analysed the intermolecular hydrogen bonds formed between the best poses of wild and alternate variant *IL10* promoters with CEBPA. Interestingly, in the case of wild‐type *IL10* promoter fragments, 18 amino acid residues of CEBPA were involved in the direct interactions with the DNA in comparison to 17 amino acid residues in the case of alternate variant form. Details of intermolecular hydrogen bonds between CEBPA and *IL10* promoter fragments are provided in Supplementary Table S1.

Similarly, we found that both the wild (‘A’ allele) and alternate variant (‘G’ allele) *IL10* dsDNA fragments containing the rs1800896 polymorphism interact with the KLF4 transcription factor (Figure 4b). In contrast to the rs1800871 genomic variants, our results suggest that the rs1800896 alternate variant dsDNA fragment (‘G’ allele) has more binding affinity with the KLF4 (binding energy: −188.596 kcal/mol) in comparison to its wild‐type (‘A’ allele) counterpart (binding energy: −186.480 kcal/mol). Analysis of intermolecular hydrogen bonds indicates that there is no change in the number of bonds formed between KLF4 and *IL10* promoter fragments. However, the bond distances are slightly reduced in the case of the *IL10* alternate variant fragment. Details of intermolecular hydrogen bonds between KLF4 and *IL10* promoter fragments are provided in Table [Link], [Link].

## 4 Discussion

Diabetic nephropathy is a microvascular complication that is caused by both environmental and genetic factors in diabetic individuals [21, 31, 32, 33]. It is distinguished by high serum creatinine and uric acid levels, reduced glomerular filtration rate, urine albumin production, glomerular lesions, and tubular fibrosis [34]. DN is increasingly prevalent and poses a major global healthcare challenge. Genetic predisposition is considered a key factor in DN pathogenesis, and various studies have implicated cytokine dysregulation in disease progression [33, 35, 36, 37]. *IL10* is an anti‐inflammatory cytokine that plays a dual role in DN pathophysiology. Altered levels of *IL10* have been associated with both protective and pathogenic effects in DN. The study of Sinuani et al., 2013 group shows elevated levels of *IL10* play a role in inflammation‐induced damage [8]. Moreover, a reduced level of *IL10* promotes mesangial immune complex deposition, contributing to persistent glomerular injury and albuminuria [9, 38].

However, the precise molecular mechanisms of *IL10* in DN progression are unclear. Previous studies have demonstrated that several SNPs in the *IL10* gene may influence cytokine expression and DN susceptibility [39]. However, findings have varied across different populations, highlighting the importance of ethnic and genetic background in determining SNP effects [12, 40, 41]. It is widely known that ethnicity has a significant influence on the distribution of allele frequencies of cytokine polymorphisms [42].

In our study, we identified a higher frequency of the homozygous alternate variant ‘CC’ genotype for *IL10* rs1800871 and the ‘GG’ genotype for *IL10* rs1800896 in DN patients compared to HC. The carriage rate frequency also increases in DN, suggesting a potential genetic susceptibility.

Individual genetic changes have a little influence on illness development but when many distinct SNPs are considered together, they can significantly expand the range of disease manifestation [43]. A haplotype analysis was conducted to assess the combined effect of *IL10* polymorphisms rs1800871 (T/C) and rs1800896 (A/G) on DN progression. The resultant data suggest that the ‘CG’ and ‘TG’ haplotypes are most susceptible for DN and T2DM. Similarly, the ‘CA’ haplotype shows an association with T2DM.

At the expression level, we observed that the heterozygous alternate variant ‘TC’ and homozygous alternate variant ‘CC’ genotypes at rs1800871 were linked to significantly reduced *IL10* mRNA expression in DN patients, compared to HC with homozygous wild‐type ‘TT’ genotype. This suggests that the ‘C’ allele may downregulate *IL10* expression, potentially contributing to disease progression. Our structural analysis suggests that the CEBPA TF binding site includes rs1800871 genomic location [44, 45]. The molecular docking analyses indicate that the alternate variant *IL10* promoter fragment has less binding affinity for the CEBPA TF compared to the wild‐type fragment. As CEBPA transactivates the *IL10* gene, the low binding affinity with the alternate variant promoter fragment might be the reason for the low *IL10* expression profile in both T2DM and DN patients having rs1800871 CC genotype compared to the wild‐type TT genotype.

Conversely, the rs1800896 A/G polymorphism was associated with increased *IL10* mRNA levels in DN patients, with both the ‘AG’ and ‘GG’ genotypes compared to the wild‐type homozygous ‘AA’ genotype. Screening of *IL10* and in vivo fragments from the ChIP‐Chip assay dataset available on TRANSFAC and prediction using the Match algorithm suggest KLF4 as a potential TF that may bind to the genomic location containing the rs1800896 mutation. Interestingly, our molecular docking analyses also indicate that the alternate variant *IL10* promoter fragment has more binding affinity with KLF4 in comparison to the wild‐type fragment [24]. These results suggest that the homozygous alternate variant ‘GG’ genotype enhances KLF4 binding to the *IL10* promoter, resulting in increased *IL10* expression compared to a wild‐type AA genotype. This finding also correlates with the *IL10* expression profile in both T2DM and DN patients containing the homozygous wild‐type ‘AA’ genotype, heterozygous ‘AG’ genotype, and homozygous alternate variant ‘GG’ genotype as shown in Figure 2C.

Our study also highlights several limitations, including a relatively small sample size and limited funding, which may have constrained the duration and scope of the study. Despite these limitations, our findings underscore the importance of the *IL10* rs1800871 and rs1800896 polymorphism in DN susceptibility and provide valuable insights into their potential use as prognostic markers in the North Indian population.

## 5 Conclusion

Our study demonstrates a significant association between *IL10* gene polymorphism (rs1800896) and the risk of DN. Our findings also offer novel insights into the transcriptional regulation of *IL10* in DN patients, suggesting that these polymorphisms may modulate *IL10* expression via altered interactions with transcription factors such as CEBPA and KLF4. These results support the potential of *IL10* gene variants as biomarkers for DN and contribute to the growing understanding of genetic influences on DN susceptibility in ethnically diverse populations. Further study on protein‐level validation of *IL10* and chromatin immunoprecipitation (ChiP) assays to confirm TF binding in patient samples would further strengthen the mechanistic conclusion.

## 6 Clinical Perspectives

- This study identifies *IL10* polymorphism rs1800896 as significantly associated with DN, alongside the ‘CG’ and ‘TG’ haplotypes, also linked to disease susceptibility in the North Indian population.
- A genetic correlation study shows that *IL10* gene expression was upregulated in the rs1800896 alternate variant genotype (GG) and downregulated in the rs1800871 alternate variant genotype (CC).
- *In silico* analyses suggest that the binding affinity of transcription factor CEBPA decreases due to the rs1800871 alternate variant while the affinity of KLF4 increases in the case of the rs1800896 alternate variant. Our *in silico* results also correlated with *IL10* expression analysis in respective patient groups. Our study highlights the prognostic significance of *IL10* gene polymorphism in DN and elucidates its regulatory role in disease pathogenesis in the North Indian population.

## Author Contributions

**Neha Shukla:** conceptualization (lead), data curation (lead), formal analysis (lead), investigation (lead), methodology (lead), software (lead), validation (lead), visualization (lead), writing – original draft (lead), writing – review and editing (lead). **Shivani Kumari:** methodology (supporting). **Poornima Verma:** methodology (supporting). **Amisha Srivastava:** methodology (supporting). **Neelam Singh:** editing (supporting). **Gyan Manjary Rao:** writing – review and editing (supporting). **Narayan Prasad:** resources (supporting). **Sushil Gupta:** resources (supporting). **Anshika Srivastava:** conceptualization (supporting), writing – review and editing (supporting). **M. S. Ansari:** resources (equal), writing – review and editing (supporting). **Soorya Janakiraman:** formal analysis (supporting), software (supporting), visualization (supporting). **Marey Baden:** software (supporting), visualization (supporting), writing – review and editing (supporting). **Olaf Wolkenhauer:** funding acquisition (supporting), resources (supporting), writing – review and editing (supporting). **Shailendra K. Gupta:** formal analysis (supporting), funding acquisition (supporting), investigation (equal), methodology (supporting), resources (supporting), software (equal), visualization (supporting), writing – review and editing (equal). **Naveen Kumar Gautam:** conceptualization (lead), data curation (supporting), formal analysis (supporting), funding acquisition (lead), investigation (equal), methodology (lead), project administration (lead), resources (lead), software (lead), supervision (lead), validation (lead), visualization (lead), writing – review and editing (lead).

## Disclosure

The authors have nothing to report.

## Conflicts of Interest

The authors declare no conflicts of interest.

## Supporting information


**Table S1:** Intermolecular Hydrogen‐bonds between CEBPA transcription factors and wild‐type/Alternate variant *IL10* promoter fragments (double‐stranded DNA around rs1800871 genomic variants).

**Table S2:** Intermolecular Hydrogen‐bonds between KLF4 transcription factors and wild‐type/alternate variant *IL10* promoter fragments (double stranded DNA around rs1800896 genomic variants).

## Data Availability

The original study data sets, supporting the findings of this study, are stored within the Sanjay Gandhi Post Graduate Institute of Medical Sciences, Lucknow, India as per the standard operating procedures of the SGPGIMS, Lucknow, India Clinical Research Unit. The data can be made available if requested via the data‐sharing processes in agreement with the study's chief investigator.
