# Supplementary material for: Exploring the Prognostic Significance of IL10 Variants and Their Mechanistic Regulation in Diabetic Nephropathy
Source: J Cell Mol Med. 2025 Sep 11;29(17):e70819. doi: 10.1111/jcmm.70819 (PMC12425813; doi:10.1111/jcmm.70819)
Supplement: Supplementary file 1 — Table S1: Intermolecular Hydrogen‐bonds between CEBPA transcription factors and wild‐type/Alternate variant IL10 promoter fragments (double‐stranded DNA around rs1800871 genomic variants). [file JCMM-29-e70819-s001.docx]

| **Supplementary Table 1: Intermolecular Hydrogen-bonds between CEBPA transcription factors and wild-type/ Alternate variant *IL10* promoter fragments (double-stranded DNA around rs1800871 genomic variants)** | | | | | | | | | |
| --- | --- | --- | --- | --- | --- | --- | --- | --- | --- |
| **Wild-type *IL10* promoter (with 'T' allele)** | |  |  |  |  |  |  |  |  |
| **Name** | **Distance** | **Category** | **Types** | **From** | **From Chemistry** | **To** | **To Chemistry** | **Angle XDA** | **Angle DAY** |
| A:ARG286:NH2 - D:DT1:O1P | 2.76727 | Hydrogen Bond;Electrostatic | Salt Bridge | A:ARG286:NH2 | H-Donor | D:DT1:O1P | H-Acceptor | 176.373 | 146.562 |
| A:ARG288:NH1 - C:DA-6:O2P | 1.55562 | Hydrogen Bond;Electrostatic | Salt Bridge | A:ARG288:NH1 | H-Donor | C:DA-6:O2P | H-Acceptor | 140.273 | 131.46 |
| A:ARG289:NH2 - D:DT1:O2P | 2.4138 | Hydrogen Bond;Electrostatic | Salt Bridge | A:ARG289:NH2 | H-Donor | D:DT1:O2P | H-Acceptor | 117.827 | 97.972 |
| A:ARG291:NH2 - C:DA-6:O1P | 3.41633 | Hydrogen Bond;Electrostatic | Salt Bridge | A:ARG291:NH2 | H-Donor | C:DA-6:O1P | H-Acceptor | 133.579 | 135.781 |
| A:LYS298:NZ - C:DA-4:O1P | 3.02664 | Hydrogen Bond;Electrostatic | Salt Bridge | A:LYS298:NZ | H-Donor | C:DA-4:O1P | H-Acceptor | 122.405 | 101.928 |
| A:LYS298:NZ - C:DA-4:O2P | 3.28174 | Hydrogen Bond;Electrostatic | Salt Bridge | A:LYS298:NZ | H-Donor | C:DA-4:O2P | H-Acceptor | 96.023 | 91.401 |
| A:LYS302:NZ - C:DG-3:O2P | 3.03848 | Hydrogen Bond;Electrostatic | Salt Bridge | A:LYS302:NZ | H-Donor | C:DG-3:O2P | H-Acceptor | 142.381 | 128.17 |
| B:ARG286:NH2 - C:DT3:O1P | 2.73791 | Hydrogen Bond;Electrostatic | Salt Bridge | B:ARG286:NH2 | H-Donor | C:DT3:O1P | H-Acceptor | 136.361 | 113.531 |
| B:ARG289:NH1 - C:DA4:O2P | 3.32734 | Hydrogen Bond;Electrostatic | Salt Bridge | B:ARG289:NH1 | H-Donor | C:DA4:O2P | H-Acceptor | 114.658 | 122.602 |
| B:ARG297:NH1 - C:DT2:O2P | 2.0036 | Hydrogen Bond;Electrostatic | Salt Bridge | B:ARG297:NH1 | H-Donor | C:DT2:O2P | H-Acceptor | 163.752 | 121.04 |
| B:LYS298:NZ - D:DG-6:O1P | 2.43535 | Hydrogen Bond;Electrostatic | Salt Bridge | B:LYS298:NZ | H-Donor | D:DG-6:O1P | H-Acceptor | 111.558 | 141.205 |
| B:LYS304:NZ - C:DT-1:O1P | 2.78387 | Hydrogen Bond;Electrostatic | Salt Bridge | B:LYS304:NZ | H-Donor | C:DT-1:O1P | H-Acceptor | 101.335 | 111.645 |
| B:ARG306:NH2 - D:DT-5:O1P | 3.54817 | Hydrogen Bond;Electrostatic | Salt Bridge | B:ARG306:NH2 | H-Donor | D:DT-5:O1P | H-Acceptor | 104.642 | 126.443 |
| A:ARG289:NH2 - D:DT1:O5' | 2.47055 | Hydrogen Bond | Conventional Hydrogen Bond | A:ARG289:NH2 | H-Donor | D:DT1:O5' | H-Acceptor | 112.591 | 92.778 |
| A:ASN293:ND2 - D:DT1:O2P | 2.29855 | Hydrogen Bond | Conventional Hydrogen Bond | A:ASN293:ND2 | H-Donor | D:DT1:O2P | H-Acceptor | 98.319 | 148.934 |
| A:ARG300:NH2 - C:DT-1:O4 | 2.37974 | Hydrogen Bond | Conventional Hydrogen Bond | A:ARG300:NH2 | H-Donor | C:DT-1:O4 | H-Acceptor | 133.615 | 127.289 |
| B:TYR285:OH - C:DA4:O2P | 3.30401 | Hydrogen Bond | Conventional Hydrogen Bond | B:TYR285:OH | H-Donor | C:DA4:O2P | H-Acceptor | 114.845 | 91.944 |
| B:ARG289:NE - C:DT3:O2P | 3.03556 | Hydrogen Bond | Conventional Hydrogen Bond | B:ARG289:NE | H-Donor | C:DT3:O2P | H-Acceptor | 109.476 | 114.877 |
| B:ASN293:ND2 - C:DT3:O2P | 2.887 | Hydrogen Bond | Conventional Hydrogen Bond | B:ASN293:ND2 | H-Donor | C:DT3:O2P | H-Acceptor | 90.115 | 150.22 |
| B:SER299:OG - D:DT-5:O2P | 2.5725 | Hydrogen Bond | Conventional Hydrogen Bond | B:SER299:OG | H-Donor | D:DT-5:O2P | H-Acceptor | 110.804 | 143.274 |
| B:ARG300:NH1 - C:DT2:O4 | 2.30601 | Hydrogen Bond | Conventional Hydrogen Bond | B:ARG300:NH1 | H-Donor | C:DT2:O4 | H-Acceptor | 102.924 | 106.448 |
| **Alternate variant *IL10* promoter (with 'C' allele)** | |  |  |  |  |  |  |  |  |
| A:ARG286:NH2 - D:DT1:O1P | 2.7699 | Hydrogen Bond;Electrostatic | Salt Bridge | A:ARG286:NH2 | H-Donor | D:DT1:O1P | H-Acceptor | 176.429 | 146.642 |
| A:ARG288:NH1 - C:DA-6:O2P | 1.56045 | Hydrogen Bond;Electrostatic | Salt Bridge | A:ARG288:NH1 | H-Donor | C:DA-6:O2P | H-Acceptor | 140.528 | 131.723 |
| A:ARG289:NH2 - D:DT1:O2P | 2.41936 | Hydrogen Bond;Electrostatic | Salt Bridge | A:ARG289:NH2 | H-Donor | D:DT1:O2P | H-Acceptor | 117.871 | 97.754 |
| A:ARG291:NH2 - C:DA-6:O1P | 3.40921 | Hydrogen Bond;Electrostatic | Salt Bridge | A:ARG291:NH2 | H-Donor | C:DA-6:O1P | H-Acceptor | 133.482 | 135.809 |
| A:LYS298:NZ - C:DA-4:O1P | 3.01885 | Hydrogen Bond;Electrostatic | Salt Bridge | A:LYS298:NZ | H-Donor | C:DA-4:O1P | H-Acceptor | 122.507 | 101.774 |
| A:LYS298:NZ - C:DA-4:O2P | 3.27122 | Hydrogen Bond;Electrostatic | Salt Bridge | A:LYS298:NZ | H-Donor | C:DA-4:O2P | H-Acceptor | 96.046 | 91.358 |
| A:LYS302:NZ - C:DG-3:O2P | 3.02995 | Hydrogen Bond;Electrostatic | Salt Bridge | A:LYS302:NZ | H-Donor | C:DG-3:O2P | H-Acceptor | 142.278 | 128.161 |
| B:ARG286:NH2 - C:DT3:O1P | 2.74416 | Hydrogen Bond;Electrostatic | Salt Bridge | B:ARG286:NH2 | H-Donor | C:DT3:O1P | H-Acceptor | 136.483 | 113.73 |
| B:ARG289:NH1 - C:DA4:O2P | 3.32582 | Hydrogen Bond;Electrostatic | Salt Bridge | B:ARG289:NH1 | H-Donor | C:DA4:O2P | H-Acceptor | 114.813 | 122.677 |
| B:ARG297:NH1 - C:DT2:O2P | 2.00782 | Hydrogen Bond;Electrostatic | Salt Bridge | B:ARG297:NH1 | H-Donor | C:DT2:O2P | H-Acceptor | 163.469 | 120.761 |
| B:LYS298:NZ - D:DG-6:O1P | 2.44024 | Hydrogen Bond;Electrostatic | Salt Bridge | B:LYS298:NZ | H-Donor | D:DG-6:O1P | H-Acceptor | 111.431 | 141.109 |
| B:LYS304:NZ - C:DT-1:O1P | 2.78245 | Hydrogen Bond;Electrostatic | Salt Bridge | B:LYS304:NZ | H-Donor | C:DT-1:O1P | H-Acceptor | 101.114 | 111.779 |
| B:ARG306:NH2 - D:DT-5:O1P | 3.54466 | Hydrogen Bond;Electrostatic | Salt Bridge | B:ARG306:NH2 | H-Donor | D:DT-5:O1P | H-Acceptor | 104.721 | 126.279 |
| A:ARG289:NH2 - D:DT1:O5' | 2.46499 | Hydrogen Bond | Conventional Hydrogen Bond | A:ARG289:NH2 | H-Donor | D:DT1:O5' | H-Acceptor | 112.752 | 93.026 |
| A:ASN293:ND2 - D:DT1:O2P | 2.28895 | Hydrogen Bond | Conventional Hydrogen Bond | A:ASN293:ND2 | H-Donor | D:DT1:O2P | H-Acceptor | 98.247 | 148.916 |
| A:ARG300:NH2 - C:DT-1:O4 | 2.37283 | Hydrogen Bond | Conventional Hydrogen Bond | A:ARG300:NH2 | H-Donor | C:DT-1:O4 | H-Acceptor | 133.401 | 127.204 |
| A:ARG300:NH2 - C:DG1:O6 | 2.55601 | Hydrogen Bond | Conventional Hydrogen Bond | A:ARG300:NH2 | H-Donor | C:DG1:O6 | H-Acceptor | 146.557 | 162.782 |
| B:TYR285:OH - C:DA4:O2P | 3.31447 | Hydrogen Bond | Conventional Hydrogen Bond | B:TYR285:OH | H-Donor | C:DA4:O2P | H-Acceptor | 114.864 | 91.987 |
| B:ARG289:NE - C:DT3:O2P | 3.0303 | Hydrogen Bond | Conventional Hydrogen Bond | B:ARG289:NE | H-Donor | C:DT3:O2P | H-Acceptor | 109.312 | 114.829 |
| B:SER299:OG - D:DT-5:O2P | 2.58016 | Hydrogen Bond | Conventional Hydrogen Bond | B:SER299:OG | H-Donor | D:DT-5:O2P | H-Acceptor | 110.972 | 143.389 |
| B:ARG300:NH1 - C:DT2:O4 | 2.30473 | Hydrogen Bond | Conventional Hydrogen Bond | B:ARG300:NH1 | H-Donor | C:DT2:O4 | H-Acceptor | 102.657 | 106.18 |
| B:ARG289:CD - C:DA4:O2P | 3.40885 | Hydrogen Bond | Carbon Hydrogen Bond | B:ARG289:CD | H-Donor | C:DA4:O2P | H-Acceptor | 107.21 | 133.975 |
| Chain A and B: CEBPA |  |  |  |  |  |  |  |  |  |
| Chain C: Forward DNA strand |  |  |  |  |  |  |  |  |  |
| Chain D: Reverse DNA strand |  |  |  |  |  |  |  |  |  |
