# Supplementary material for: Exploring the Prognostic Significance of IL10 Variants and Their Mechanistic Regulation in Diabetic Nephropathy
Source: J Cell Mol Med. 2025 Sep 11;29(17):e70819. doi: 10.1111/jcmm.70819 (PMC12425813; doi:10.1111/jcmm.70819)
Supplement: Supplementary file 2 — Table S2: Intermolecular Hydrogen‐bonds between KLF4 transcription factors and wild‐type/alternate variant IL10 promoter fragments (double stranded DNA around rs1800896 genomic variants). [file JCMM-29-e70819-s002.docx]

| **Supplementary Table 2: Intermolecular Hydrogen-bonds between KLF4 transcription factors and wild-type/ alternate variant *IL10* promoter fragments (double stranded DNA around rs1800896 genomic variants)** | | | | | | | | | |
| --- | --- | --- | --- | --- | --- | --- | --- | --- | --- |
| **Wild-type *IL10* promoter (with 'A' allele)** | |  |  |  |  |  |  |  |  |
| **Name** | **Distance** | **Category** | **Types** | **From** | **From Chemistry** | **To** | **To Chemistry** | **Angle XDA** | **Angle DAY** |
| A:ARG473:NH1 - C:DC9:O2P | 2.1995 | Hydrogen Bond;Electrostatic | Salt Bridge | A:ARG473:NH1 | H-Donor | C:DC9:O2P | H-Acceptor | 105.249 | 111.37 |
| A:ARG473:NH1 - C:DC10:O1P | 3.53716 | Hydrogen Bond;Electrostatic | Salt Bridge | A:ARG473:NH1 | H-Donor | C:DC10:O1P | H-Acceptor | 133.409 | 107.862 |
| A:ARG473:NH2 - B:DG7:O1P | 3.3943 | Hydrogen Bond;Electrostatic | Salt Bridge | A:ARG473:NH2 | H-Donor | B:DG7:O1P | H-Acceptor | 155.89 | 111.398 |
| A:ARG479:NH1 - C:DC8:O2P | 3.29006 | Hydrogen Bond;Electrostatic | Salt Bridge | A:ARG479:NH1 | H-Donor | C:DC8:O2P | H-Acceptor | 116.747 | 146.391 |
| A:SER445:OG - B:DT5:O2P | 1.73365 | Hydrogen Bond | Conventional Hydrogen Bond | A:SER445:OG | H-Donor | B:DT5:O2P | H-Acceptor | 139.215 | 118.634 |
| A:HIS446:N - B:DT5:O2P | 3.12386 | Hydrogen Bond | Conventional Hydrogen Bond | A:HIS446:N | H-Donor | B:DT5:O2P | H-Acceptor | 96.368 | 162.373 |
| A:HIS446:ND1 - C:DT11:O1P | 3.03451 | Hydrogen Bond | Conventional Hydrogen Bond | A:HIS446:ND1 | H-Donor | C:DT11:O1P | H-Acceptor | 99.932 | 109.749 |
| A:SER502:OG - B:DG8:O3' | 2.04244 | Hydrogen Bond | Conventional Hydrogen Bond | A:SER502:OG | H-Donor | B:DG8:O3' | H-Acceptor | 137.563 | 92.669 |
| B:DT5:C5' - A:SER445:OG | 2.44024 | Hydrogen Bond | Carbon Hydrogen Bond | B:DT5:C5' | H-Donor | A:SER445:OG | H-Acceptor | 97.378 | 105.097 |
| A:HIS446:CD2 - B:DT5:O2P | 2.70347 | Hydrogen Bond | Carbon Hydrogen Bond | A:HIS446:CD2 | H-Donor | B:DT5:O2P | H-Acceptor | 100.299 | 117.712 |
| **Alternate variant *IL10* promoter (with 'G' allele)** | |  |  |  |  |  |  |  |  |
| A:ARG473:NH1 - C:DC9:O2P | 2.202 | Hydrogen Bond;Electrostatic | Salt Bridge | A:ARG473:NH1 | H-Donor | C:DC9:O2P | H-Acceptor | 105.092 | 111.131 |
| A:ARG473:NH1 - C:DC10:O1P | 3.52985 | Hydrogen Bond;Electrostatic | Salt Bridge | A:ARG473:NH1 | H-Donor | C:DC10:O1P | H-Acceptor | 133.395 | 107.932 |
| A:ARG473:NH2 - B:DG7:O1P | 3.39196 | Hydrogen Bond;Electrostatic | Salt Bridge | A:ARG473:NH2 | H-Donor | B:DG7:O1P | H-Acceptor | 155.805 | 111.559 |
| A:ARG479:NH1 - C:DC8:O2P | 3.28515 | Hydrogen Bond;Electrostatic | Salt Bridge | A:ARG479:NH1 | H-Donor | C:DC8:O2P | H-Acceptor | 116.61 | 146.268 |
| A:SER445:OG - B:DT5:O2P | 1.72448 | Hydrogen Bond | Conventional Hydrogen Bond | A:SER445:OG | H-Donor | B:DT5:O2P | H-Acceptor | 139.231 | 118.716 |
| A:HIS446:N - B:DT5:O2P | 3.12171 | Hydrogen Bond | Conventional Hydrogen Bond | A:HIS446:N | H-Donor | B:DT5:O2P | H-Acceptor | 96.203 | 162.262 |
| A:HIS446:ND1 - C:DC11:O1P | 3.02769 | Hydrogen Bond | Conventional Hydrogen Bond | A:HIS446:ND1 | H-Donor | C:DC11:O1P | H-Acceptor | 99.973 | 109.813 |
| A:SER502:OG - B:DG8:O3' | 2.03516 | Hydrogen Bond | Conventional Hydrogen Bond | A:SER502:OG | H-Donor | B:DG8:O3' | H-Acceptor | 137.734 | 92.743 |
| B:DT5:C5' - A:SER445:OG | 2.44093 | Hydrogen Bond | Carbon Hydrogen Bond | B:DT5:C5' | H-Donor | A:SER445:OG | H-Acceptor | 97.106 | 104.931 |
| A:HIS446:CD2 - B:DT5:O2P | 2.70687 | Hydrogen Bond | Carbon Hydrogen Bond | A:HIS446:CD2 | H-Donor | B:DT5:O2P | H-Acceptor | 100.407 | 117.584 |
| Chain A: KLF4 |  |  |  |  |  |  |  |  |  |
| Chain B: Forward DNA strand |  |  |  |  |  |  |  |  |  |
| Chain C: Reverse DNA strand |  |  |  |  |  |  |  |  |  |
